# Japanese Encephalitis Virus exploits the microRNA-432 to regulate the expression of Suppressor of Cytokine Signaling (SOCS) 5

**DOI:** 10.1038/srep27685

**Published:** 2016-06-10

**Authors:** Nikhil Sharma, Kanhaiya L. Kumawat, Meghana Rastogi, Anirban Basu, Sunit K. Singh

**Affiliations:** 1 Laboratory of Neurovirology and Inflammation Biology, CSIR-Centre for Cellular and Molecular Biology (CCMB), Uppal Road, Hyderabad-500007, India; 2 National Brain Research Centre Manesar, Haryana-122050, Haryana, India; 3 Laboratory of Human Molecular Virology & Immunology, Molecular Biology Unit, Faculty of Medicine, Institute of Medical Sciences (IMS), Banaras Hindu University (BHU), Varanasi-221005, India

## Abstract

Japanese encephalitis virus (JEV) is a plus strand RNA virus, which infects brain. MicroRNAs are regulatory non-coding RNAs which regulate the expression of various genes in cells. Viruses modulate the expression of various microRNAs to suppress anti-viral signaling and evade the immune response. SOCS (Suppressor of cytokine signalling) family of proteins are negative regulators of anti-viral Jak-STAT pathway. In this study, we demonstrated the regulatory role of SOCS5 in Jak-STAT signaling and its exploitation by JEV through a microRNA mediated mechanism. JEV infection in human brain microglial cells (CHME3) downregulated the expression of miR-432, and upregulated SOCS5 levels. SOCS5 was validated as a target of miR-432 by using 3′UTR clone of SOCS5 in luciferase vector along with miR-432 mimic. The overexpression of miR-432 prior to JEV infection enhanced the phosphorylation of STAT1 resulting into increased ISRE activity and cellular inflammatory response resulting into diminished viral replication. The knockdown of SOCS5 resulted into increased STAT1 phosphorylation and suppressed viral replication. JEV infection mediated downregulation of miR-432 leads to SOCS5 upregulation, which helps the virus to evade cellular anti-viral response. This study demonstrated that JEV utilizes this microRNA mediated strategy to manipulate cellular immune response promoting JEV pathogenesis.

Japanese encephalitis virus (JEV) is a mosquito borne plus strand RNA virus which infects CNS and resides in neuronal and microglial cells. JEV is a neurotropic virus causing neuroinflammation and neuronal damage^1^. It mainly infects children between 1–5 years of age and leads to the permanent neuronal damage, motor deficits and memory loss^2^. JEV has adopted a zoonotic life cycle between pigs, water birds and *Culex* mosquitoes. Human are dead end host for the virus^3^. In spite of vaccines available against JEV, three million deaths have been estimated due to lack of effective anti-viral drug against JEV^4^. JEV is able to modulate the anti-viral immune signalling pathways to dampen the cellular anti-viral response.

MicroRNAs are small RNAs (19–24 nucleotides) regulating the expression of about 60% of human genes by binding through their seed region to the complementary sites present in 3′UTR of target gene^5^. Viruses have been reported to modulate the expression pattern of cellular microRNAs either to suppress immune response or to increase the rate of replication^6^,^7^. Viral proteins have also been reported to modulate miRNA expression. Bakre *et al*.^8^, reported that respiratory syncytial virus (RSV) NS1 protein interacts with KLF6 transcription factor to modulate miR-24 expression which facilitates viral replication^8^. JEV has been reported to induce the expression of miR-15b which targets ring finger protein RNF125, a negative regulator of RIG-1 pathway^9^. We have also reported the miR-146a mediated regulation of anti-viral Jak-STAT pathway, by JEV to promote its survival^10^.

The expression of interferon (IFN) during viral Infections triggers the anti-viral Jak-STAT signaling (Jak- Janus kinases; STAT- Signal transducer and activator of transcription) in the infected cells. Interferon binds to IFN-α/β receptors, which recruits Janus kinases (Jak). Jak autophosphorylates itself and activates the STATs (Signal Transducer and Activator of Transcription) by phosphorylation^11^. STATs undergo phosphorylation and dimerization and bind to IRF-9 to form ISGF-3 complex (Interferon Stimulated Gene Factor-3) which enters in nucleus to bind ISRE elements present at promoter region of Interferon Stimulated genes^12^. This anti-viral mechanism of the infected cells is negatively regulated by SOCS (Suppressor of Cytokine Signaling) family of proteins which include various members like SOCS1, SOCS2, SOCS3, and SOCS5^13^. SOCS1 and SOCS3 are well studied inhibitory proteins of Jak-STAT pathway. Viruses have been reported to induce the expression of SOCS proteins to suppress cellular anti-viral response^14^. Linossi *et al*. demonstrated that SOCS5 interacts with Jak (Janus Kinases) via its Jak interacting region (JIR) and inhibits the auto-phosphorylation of Jak1 and Jak2^15^. This leads to the inhibition of the kinase activity of Jak and hence, SOCS5 negatively regulate Jak-STAT signalling. However, the function of SOCS5 has not been well studied with respect to the viral infections. miR-432 has been reported to activate Wnt/β-catenin signalling and its downregulation in hepatocytes promoted hepatocellular carcinoma^16^. Chen *et al*. has also demonstrated tumor suppressive role of miR-432 in lung cancer cells^17^. The role of miR-432 in modulating immune signalling pathways during viral infections has not been well elucidated.

In our study, we found downregulated expression of miR-432 in our microRNA profiling data in JEV infected CHME3 cells. Bioinformatics tools predicted SOCS5 as a potential target of miR-432 so we elucidated the role of microRNA mediated regulation of SOCS5 during JEV infection. We have demonstrated downregulation of miR-432 by JEV leads to SOCS5 upregulation in JEV infected CHME3 cells as well as in JEV infected mice brain. We validated SOCS5 as a target of miR-432 and demonstrated the effect of overexpression and silencing of miR-432 on ISRE activity, production of pro-inflammatory cytokines and its effect on viral replication.

## Results

### JEV infection downregulates miR-432 expression

**Figure 1:**
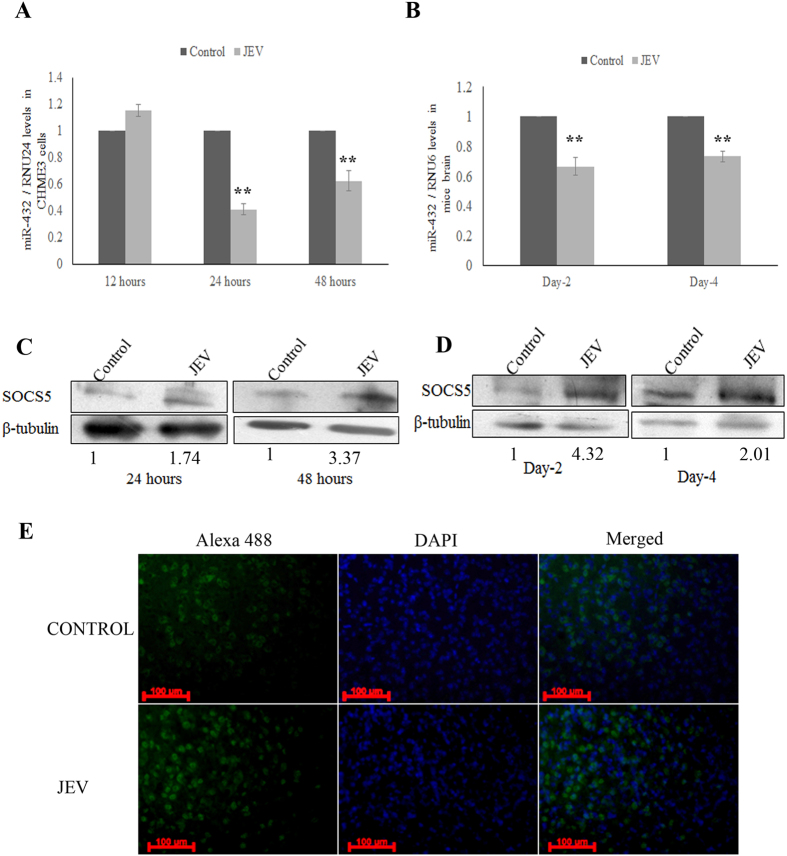
JEV infection downregulates miR-432 levels and leads to SOCS5 upregulation. Human brain microglial cells were infected by JEV (MOI 5) and cells were harvested at various time points to determine miR-432 levels. **(A)** Graph showing downregulation of miR-432 levels at 24 and 48 hours post infection as compared to 12 hours sample. TaqMan probe specific to miR-432 were used to determine fold change. The C_t_ values were normalized by RNU-24 levels. **(B)** Graph showing reduced levels of miR-432 in JEV infected mice brain. The RNA was isolated from brain tissue of 2 day and 4 day infected mice and mock infected mice brain was used as control. The C_t_ values were normalized by RNU-6 levels. **(C)** Western blots depicting upregulation of SOCS5 upon JEV infection. CHME3 cells were infected by JEV (MOI 5) and harvested after 24 and 48 hours. The average fold change values with respect to control have been mentioned. **(D)** Western blots showing upregulation of SOCS5 in JEV infected brain tissue. Mock infected mice brain tissue was used as control. Infected mice were harvested 2 day and 4 day post infection. β-tubulin was used for normalization. The average fold change values with respect to control have been mentioned. **(E)** Immunohistochemistry image showing increased levels of SOCS5 protein in brain of JEV infected BALB/c mice. Mock and JEV infected mice brain sections were collected 2 days post JEV infection. Enhanced levels of Alexa 488 florescence was observed in JEV infected brain section (Magnification 20x, Scale bar 100 μm). All experiments were performed in triplicates. The data are shown as mean ± S.E from three independent experiments. The fold change is significant where *denotes P < 0.05, **denotes P < 0.01, ***denotes P < 0.001.

JEV infection modulates cellular microRNA expression pattern, which further alters the expression of various genes. The miR-432 was found to be downregulated in CHME3 cells upon JEV infection (Fig. 1A). Replication of JEV in CHME3 cells was confirmed by real time PCR (Supplementary Fig. 2A). The validation of miR-432 downregulation upon JEV infection was also done in brain tissue of JEV infected mice *in-vivo* (Fig. 1B). Replication of JEV in mice brain was confirmed by real time PCR (Supplementary Fig. 2B). Since SOCS5 is a potential target of miR-432, therefore the levels of SOCS5 expression was analysed post JEV infection. JEV induced the SOCS5 expression levels in CHME3 cells (Fig. 1C). The seed region for mature miR-432 was found to be conserved in both human and mouse. Therefore SOCS-5 expression levels were checked in mice brain tissue by western blotting and found to be upregulated (Fig. 1D). In addition, we analysed SOCS5 expression in JEV infected mice brain by immunohistochemistry and found enhanced levels of SOCS5 in JEV infected mice brain tissue (Fig. 1E). This demonstrated that JEV downregulated miR-432 levels in mouse brain, which led SOCS5 upregulation. SOCS5 has been reported to inhibit auto-phosphorylation of Jak1^15^ which inhibits Jak-STAT pathway downstream.

### miR-432 targets SOCS5

**Figure 2:**
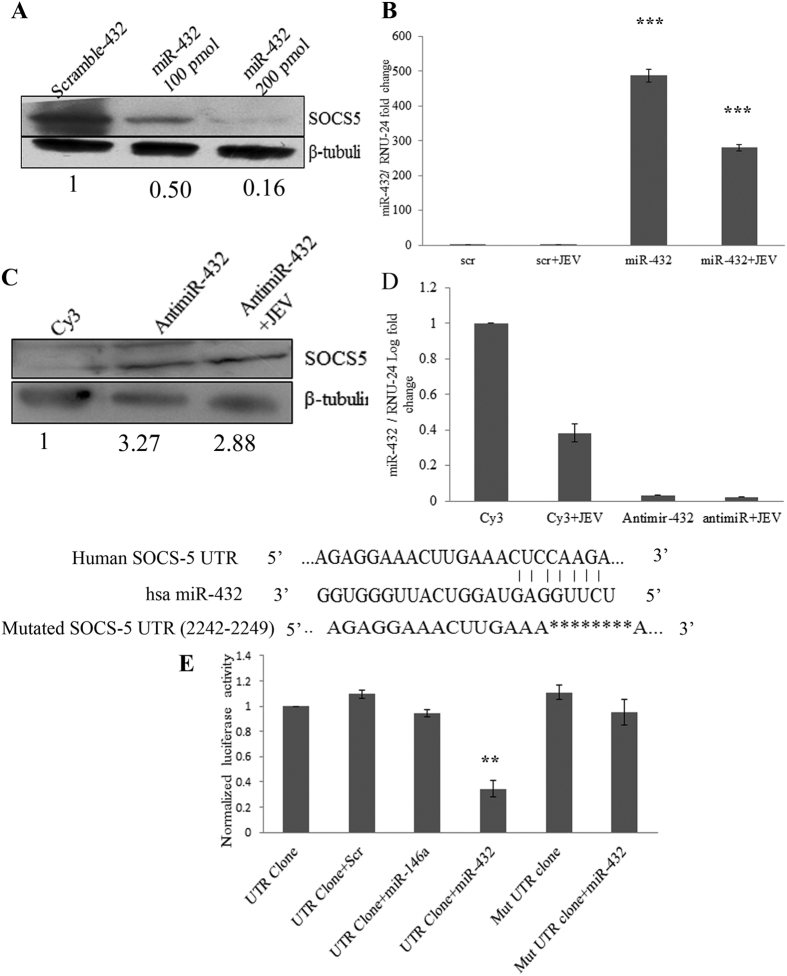
miR-432 targets SOCS5. For validation of SOCS5 UTR as target of miR-432, 100 pmol of miR-432 mimic was overexpressed in CHME3 cells. **(A)** Western blot showing downregulation of SOCS5 upon miR-432 overexpression in CHME3 cells. The average fold change values with respect to control have been mentioned. **(B)** Overexpression of miR-432 mimic was confirmed by Real time PCR using miR-432 specific TaqMan probe. RNU24 was used for normalization. **(C)** Blot showing upregulation of SOCS5 upon antimiR-432 transfection. The average fold change values with respect to control have been mentioned**. (D)** Silencing of miR-432 by antimiR-432 transfection was confirmed by real time PCR. **(E)** SOCS5 UTR clone (pEZX-MT06) for microRNA targeting was transfected along with miR-432 mimic along with β-galactosidase vector. Graph shows decreased luciferase activity of the vector in presence of miR-432 mimic. A mutant clone was generated deleting the targeting region present in SOCS5 UTR (shown in Fig. 2) and transfected along with miR-432 mimic. The mutant clone did not display any reduction in luciferase activity. Non-targeting mimic miR-146a was also used as negative control. β-galactosidase activity was used for normalization. All experiments were performed in triplicates. The data are shown as mean ± S.E from three independent experiments. The fold change is significant where *denotes P < 0.05, **denotes P < 0.01, ***denotes P < 0.001.

To confirm the targeting of SOCS5 UTR by miR-432, mimic sequence of miR-432 was overexpressed in CHME3 cells along with scramble control. The overexpression and silencing of miR-432 was confirmed by real time PCR. The SOCS5 was downregulated upon miR-432 overexpression (Fig. 2A). To further delineate the effect of miR-432 on SOCS5, antimiR-432 was overexpressed in CHME3 cells, which resulted into increased levels of SOCS5 upon antimiR-432 overexpression (Fig. 2C). To further validate the targeting of 3′UTR of SOCS5 by miR-432 mimic, luciferase vector containing 3′UTR of SOCS5 was transfected along with miR-432 mimic. The decreased luciferase activity was observed due to targeting of SOCS5 3′UTR by miR-432 (Fig. 2D). A mutant was generated by deleting the targeting site of miR-432 present in 3′UTR of SOCS5 and cloned in the luciferase vector. The mutated UTR cloned in luciferase vector did not display any reduction of luciferase activity, when transfected along with miR-432 mimic. This confirmed the targeting of 3′UTR of SOCS5 by miR-432.

### miR-432 overexpression enhances Jak-STAT signaling by downregulating SOCS5

**Figure 3:**
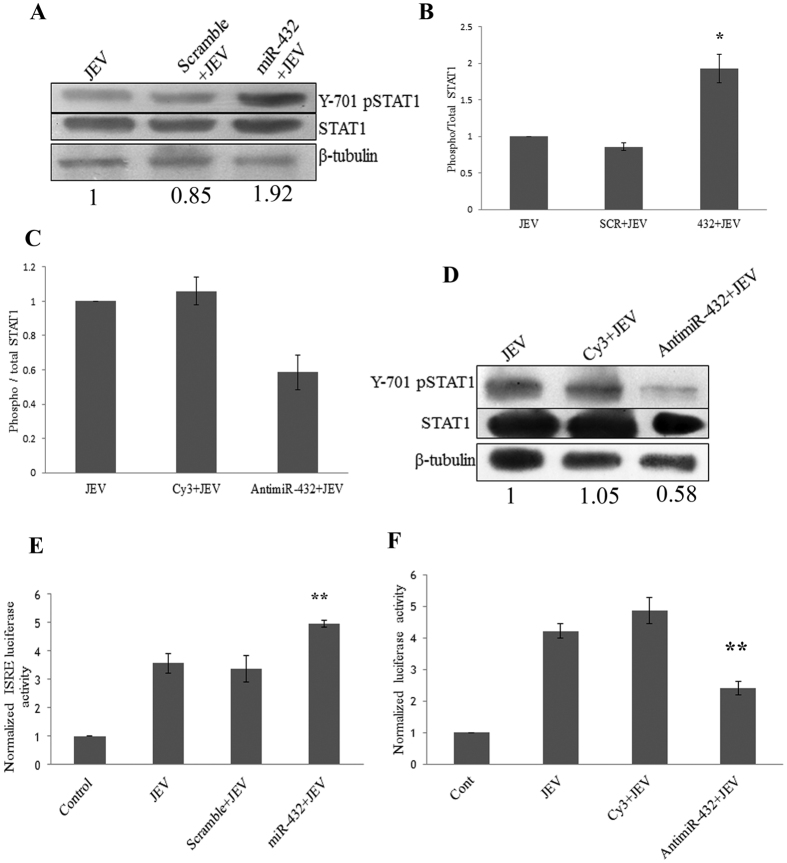
miR-432 upregulates STAT1 phosphorylation upon JEV infection. To determine the effect of miR-432 on JAK-STAT pathway, miR-432 mimic was overexpressed along with scramble sequence as control and infected by JEV 24 hours post transfection. Cells were harvested 24 hours after infection. **(A)** Western blot showing upregulation of STAT1 phosphorylation upon miR-432 overexpression prior to JEV infection as compared to scramble control. The average fold change values with respect to control have been mentioned. **(B)** Densitometry plot depicting enhanced STAT1 phosphorylation in miR-432 overexpressing cells upon JEV infection. Phospho/total STAT1 ratio was calculated which was divided by β-tubulin values. Scramble+JEV was used as control for statistical analysis. **(C)** Densitometry plot showing reduced STAT1 phosphorylation in antimiR-432 overexpressing cells upon JEV infection. Phospho/total STAT1 ratio was calculated which was divided by β-tubulin values. Cy3 labelled scramble antimiR+JEV was used as control for statistical analysis. **(D)** Western blots showing downregulation of STAT1 phosphorylation in antimiR-432 overexpressing cells upon JEV infection as compared to Cy3 labelled scramble antimiR+JEV as control. The average fold change values with respect to control have been mentioned. **(E)** ISRE luciferase vector was transfected into CHME3 cells along with miR-432 mimic or scramble sequence. JEV infection was given 24 hours post transfection and harvested at 24 hours post infection. Graph shows increased luciferase activity upon JEV infection in mir-432 transfected cells as compared to scramble transfected cells. β-galactosidase vector was used for normalization. **(F)** Graph shows reduced ISRE activity in antimiR-432 transfected cells upon JEV infection. Cy3 labelled scramble antimiR was used as control for statistical analysis. All experiments were performed in triplicates. The data are shown as mean ± S.E from three independent experiments. The fold change is significant where *denotes P < 0.05, **denotes P < 0.01, ***denotes P < 0.001.

SOCS5 is a negative regulator of Jak-STAT pathway^18^ so the effect of miR-432 overexpression was analysed on Jak-STAT pathway. Overexpression of miR-432 led to the downregulation of SOCS5 and this resulted to enhanced phosphorylation of STAT1 post JEV infection (Fig. 3A). Hence, this confirmed the negative regulatory effect of SOCS5 on Jak-STAT pathway. To further visualize the effect of miR-432 downstream STAT1 phosphorylation, the ISRE activity was visualized by luciferase vector containing ISRE sequences at promoter site. The enhanced luciferase activity was observed in miR-432 overexpressing cells post JEV infection (Fig. 3E). The increased ISRE activity was due to increased phosphorylation of STAT1. To further confirm the specificity of this effect, anti-miR-432 was transfected into cells prior to JEV infection. AntimiR-432 transfection led to the decreased phosphorylation of STAT1 (Fig. 3D) and reduced ISRE activity post-JEV infection (Fig. 3F). miR-432 downregulated SOCS5; which led to the enhanced phosphorylation of STAT1 and increased ISRE activity.

### miR-432 overexpression suppress viral replication

**Figure 4:**
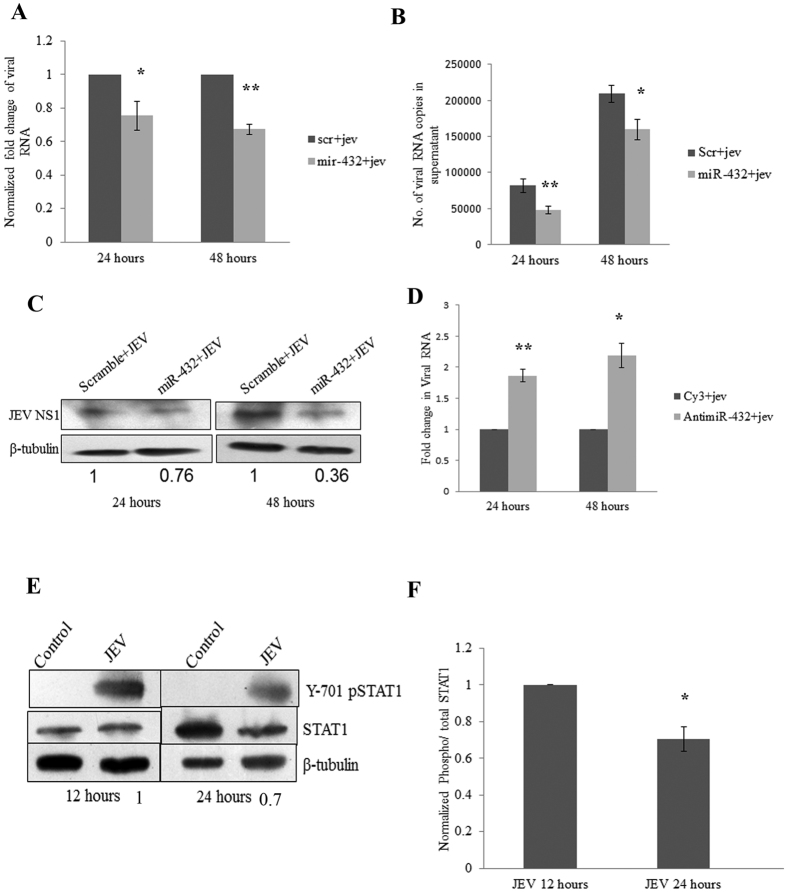
miR-432 downregulates viral replication upon JEV infection. To elucidate the effect of miR-432 on JEV replication, 100 pmol miR-432 mimic or scramble sequence was transfected into CHME3 cells and JEV infection was given 24 hours post transfection. Later cells were harvested 24 and 48 hours post infection. **(A)** Viral RNA was quantified by viral NS3 specific primers by Real time PCR. GAPDH levels were used for normalization. Graph shows downregulated levels of viral RNA in miR-432 overexpressing cells at both time points as compared to scramble transfected cells. Fold change was calculated by 2^−∆∆C^_t_ method. **(B)** Viral RNA was isolated from culture supernatant and viral RNA copies were determined by real time PCR. Copies were determined by running standard series of viral RNA by absolute quantification. miR-432 overexpression reduced viral copies in supernatant at both time points. **(C)** Western blots showing downregulation of viral NS1 protein upon miR-432 overexpression at 24 and 48 hours post infection. The average fold change values with respect to control have been mentioned. **(D)** Graph showing increased viral RNA levels in antimiR-432 transfected cells. Viral NS3 specific primers were used and GAPDH levels were used for normalization. Fold change was calculated by 2^−∆∆C^_t_ method. **(E)** Western blots depicting phosphorylation of Y-701 position of STAT1 upon JEV infection. The phosphorylation of STAT1 was higher at 12 hours post infection as compared to 24 hours after infection. The average fold change values with respect to 12 hour JEV control have been mentioned. **(F)** Densitometry plot showing phosphorylation of STAT1 at 12 hour and 24 hour post JEV infection. Phospho/total STAT1 ratio was calculated which was divided by β-tubulin values for normalization. 24 hour sample was compared to 12 hour JEV infected sample. All experiments were performed in triplicates. The data are shown as mean ± S.E from three independent experiments. The fold change is significant where *denotes P < 0.05, **denotes P < 0.01, ***denotes P < 0.001.

During the course of infection, JEV gradually increases its copy number by supressing the anti-viral machinery of the cell. Fan *et al*. has studied the kinetics of JEV replication in BHK-21 cells and found that JEV genome increases till 36 hours post infection and later attain a plateau stage^19^. Interferon secreted upon JEV infection binds to IFN-α/β receptor and leads to phosphorylation and activation of STAT1. We found decrease in STAT1 phosphorylation (Y-701) at 24 hours after JEV infection as compared to 12 hours post JEV infection (Fig. 4E). The similar pattern of STAT1 phosphorylation (Y-701) was also observed in JEV infected mice brain tissue. The phosphorylation of STAT1 decreased in day-4 post infected mice as compared to day-2 post infected mice (Supplementary Fig. 2E). The expression of Interferon stimulated genes (IFIT1 and IFIT2) also reduced with progression of infection (Supplementary Fig. 2C,D). This indicated that JEV modulates the cellular machinery to suppress anti-viral response to create a favourable milieu in the cell.

The overexpression of miR-432 resulted into the suppression of SOCS5 expression that ultimately led to the enhanced STAT1 phosphorylation creating strong anti-viral milieu in the cell due to enhanced ISRE activity. The increased levels of IL-6 and TNF-α were found in miR-432 overexpressing cells after JEV infection (Supplementary Fig. 3A,C). The viral RNA titre was found to be lower in miR-432 overexpressing cells which indicated that miR-432 suppressed the viral replication (Fig. 4A). Additionally, the viral NS1 protein was also checked through western blotting which demonstrated the decreased viral protein levels (Fig. 4C). The viral RNA was isolated from culture supernatant and viral copies were determined to check the effect of miR-432 on release of viral particles. We found decrease in viral copies in miR-432 overexpressing cells as compared to scramble transfected cells (Fig. 4B). We found enhanced viral replication in antimiR-432 transfected cells due to increased levels of SOCS5 in antimR-432 transfected cells (Fig. 4D). The increased expression of miR-432 suppressed SOCS5 levels and created strong anti-viral environment in the cell, which led the reduced viral replication.

### SOCS5 knock-down supresses viral replication

**Figure 5:**
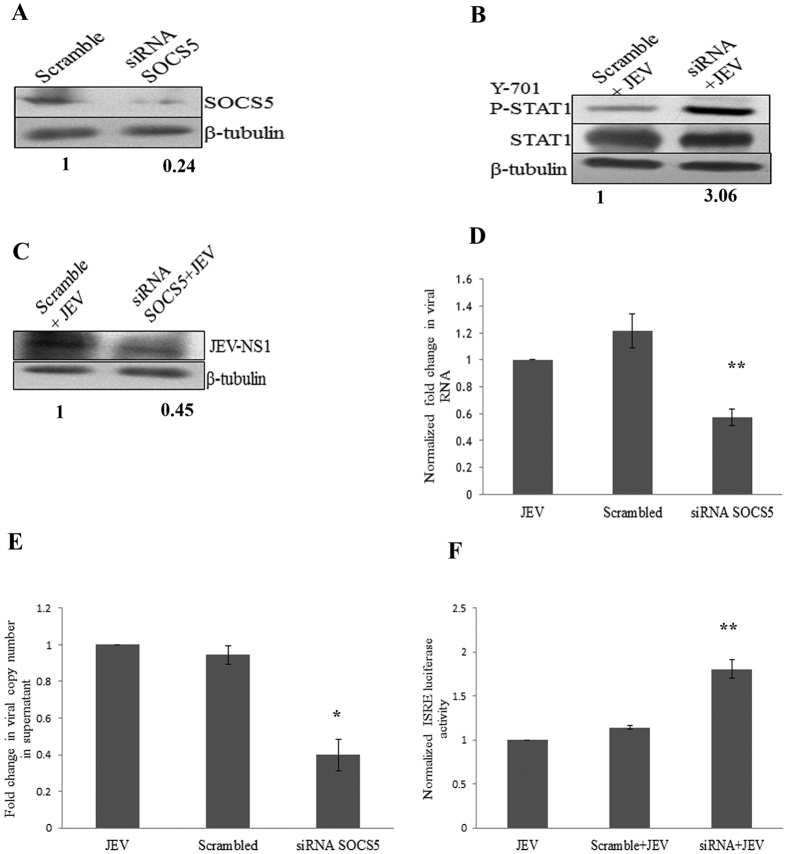
SOCS5 knock down restricts JEV replication. To decipher regulatory role of SOCS5 on JEV replication, siRNA mediated knock down of SOCS5 was done in CHME3 cells and cells were given JEV infection after 30 hours post transfection. **(A)** SOCS5 knock down was confirmed by western blotting. Scramble negative control sequence was transfected as control. The average fold change values with respect to control have been mentioned. **(B)** Western blot depicting enhanced STAT1 phosphorylation in SOCS5 knock down cells. The average fold change values with respect to control have been mentioned. **(C)** Viral NS1 protein blot showing downregulation of NS1 protein in SOCS5 knock down cells. The average fold change values with respect to control have been mentioned. **(D)** Real-time graph showing downregulation of viral RNA in SOCS5 knock down cells. RNA was isolated from the cells and viral RNA was determined by using viral NS3 specific primers. GAPDH was used for normalization. Fold change was calculated by 2^−∆∆C^_t_ method. JEV infected sample was used as control for statistical analysis. **(E)** Viral RNA was isolated from culture supernatant to determine copies of viral RNA released. The number of viral copies was calculated by running standard series of viral RNA and viral copies were determined by absolute quantification. Fold change in viral copies was calculated by assuming viral copies in supernatant of mock transfected (JEV infected) cells as control. **(F)** Graph showing enhanced ISRE activity in SOCS5 knock down cells upon JEV infection. All experiments were performed in triplicates. The data are shown as mean ± S.E from three independent experiments. The fold change is significant where *denotes P < 0.05, **denotes P < 0.01, ***denotes P < 0.001.

To further decipher the specific role of SOCS5 during JEV infection, we used siRNA mediated approach to knock-down of SOCS5 gene. 10 nM of siRNA was transfected along with scramble sequence and knock down was confirmed by western blotting (Fig. 5A). The SOCS5 silenced cells were infected by JEV to demonstrate its effect on JEV replication. We found that SOCS5 knock down enhanced the STAT1 phosphorylation which confirmed the negative regulatory effect of SOCS5 on Jak-STAT pathway (Fig. 5B). To analyse the downstream effect of SOCS5 knock down, the ISRE activity was checked in SOCS5 knocked down cells enhanced ISRE activity was found in SOCS5 knockdown cells compared to scramble transfected cells upon JEV infection (Fig. 5F). SOCS5 knockdown suppressed viral replication in CHME3 cells and resulted into decreased in viral RNA (Fig. 5D) as well as viral NS1 protein in SOCS5 knockdown cells (Fig. 5C). The release of virus in culture supernatant was also suppressed in SOCS5 knockdown cells (Fig. 5E). The SOCS5 gene was cloned in eukaryotic vector pcDNA 3.1 and SOCS5 was overexpressed in CHME3 cells prior to JEV infection. SOCS5 overexpressing cells displayed reduced expression of pro-inflammatory cytokines (IL-6 and TNF-α) (Supplementary Fig. 3B,D) and higher JEV replication (Supplementary Fig. 1B,C). The higher copies of JEV RNA as well as NS1 protein expression was observed in SOCS5 overexpressing cells compared to empty vector transfected cells. This explains that the suppressive effect of miR-432 on JEV replication is specifically due to SOCS5 downregulation in miR-432 overexpressing cells and SOCS5 has a direct regulatory effect on anti-viral Jak-STAT pathway.

## Discussion

Viruses have evolved various strategies to evade cellular immune response by exploiting the host cellular machinery. Various viral proteins interact with different anti-viral proteins to modulate their functions. Many viruses and viral proteins adopt microRNA mediated regulation of anti-viral pathways to alleviate their pathogenesis. Influenza A virus utilizes miR-302c to target NF-κB kinase activity in order to modulate IFN-β pathway^20^. Hepatitis B virus X protein has been reported to induce the expression of miR-21 to promote hepatocellular carcinoma in hepatocytes^21^. Few viral proteins of RNA viruses have been reported to localize into nucleus and interact with histones to modify gene expression^22^,^23^.

Wang *et al*.^24^ has reported the crucial role of Shp-2 (a PTP) for controlling the replication of respiratory syncytial virus (RSV) in A549 cells. They reported that the blocking of Shp-2 led to the impaired STAT1 phosphorylation^24^. JEV NS5 has been reported to block STAT1 phosphorylation by activation of phospho tyrosine phosphatases PTPs^25^. SOCS family of proteins have been well characterized as negative regulators of Jak-STAT pathway^26^,^27^. RSV non-structural proteins have been reported to induce the expression of SOCS1 and SOCS3 proteins ultimately leading to the suppression of IFN signaling^28^. Kundu *et al*.^29^ has reported the upregulation of SOCS1 and SOCS3 in JEV infected mouse macrophages^29^. Li *et al*.^30^ has reported the upregulation of SOCS3 in JEV infected mice brain^30^. The upregulation of these SOCS proteins alleviate viral survival in host cells. Kario *et al*.^31^ has reported the SOCS5 as a negative regulator of EGFR signalling^31^. Zhuang *et al*.^32^ demonstrated targeting of SOCS5 by miR-9 in vascular endothelial cells co-cultured with tumour cells which promoted endothelial cell migration^32^. However, the role of SOCS5 has not been well understood during viral infection.

JEV adopts various strategies to facilitate its replication in the host cells. JEV infection leads to the activation of microglial cells resulting into the release of pro-inflammatory cytokines^33^,^34^. JEV infection triggers antiviral Jak-STAT pathway by increasing the STAT1 phosphorylation in human microglial cells in early course of infection. The downregulation of phospho STAT1 levels was observed at the late time point (24 hours). In our previous study, we reported the decreased ISRE activity as well as reduced levels of expression of Interferon stimulated genes (ISG-56, ISG-54) during late time points of infection^10^. A similar immunosuppressive pattern of STAT1 phosphorylation was observed in JEV infected mice brain tissue. The phosphorylation of STAT1 and expression of IFIT1 and IFIT2 was reduced in 4 day post infected tissue as compared to 2 day infected tissue (Supplementary Fig. 2C–E). Hence we hypothesize that virus tries to suppress antiviral signalling at different time points of infection to evade immune response through various strategies.

We demonstrated that JEV downregulates miR-432 in CHME3 cells, which leads to the increased expression of SOCS5. To validate the SOCS5 targeting by miR-432, the UTR clone of SOCS5 in luciferase vector along with miR-432 mimic was transfected in HeLa cells, which resulted into reduced luciferase activity. To further study the specificity of the targeting, the recognition sequences of miR-432 present in 3′UTR of SOCS5 were deleted and cloned in to luciferase vector. The mutated UTR clone did not display reduction in luciferase activity which confirmed the SOCS5 as a target of miR-432. Since SOCS5 inhibits auto-phosphorylation of Jaks, the effect of miR-432 overexpression was analysed on Jak-STAT signalling. miR-432 overexpression suppressed SOCS5 levels causing enhanced Y-701 STAT1 phosphorylation. This resulted into elevated ISRE activity and upregulated expression of pro-inflammatory cytokines. miR-432 overexpression caused enhanced production of pro-inflammatory cytokines (IL-6 and TNF-α) (Supplementary Fig. 3A,C). Hence, miR-432 overexpression created a potent antiviral milieu in the cell, which further led to the reduced viral replication in the cells as well as release of viral particles in culture supernatant. Anti-miR-432 reduced STAT1 phosphorylation and lowered ISRE activity upon JEV infection, which led to the enhanced viral replication.

To further ascertain the negative regulatory effect of SOCS5 on Jak-STAT pathway, we specifically knocked down SOCS5 by using siRNA. SOCS5 knock down led to the enhanced phosphorylation of STAT1 and increased ISRE activity. siRNA mediated SOCS5 knock down strengthened the cellular anti-viral response, which led to the reduction in viral replication in knock down cells as well as diminished release of viral particles in culture supernatant. Watanabe *et al*. had deciphered the role of SOCS5 overexpressing T-cells in augmenting the release of pro-inflammatory cytokines from macrophages during septic peritonitis^35^. We also analysed the effect of JEV infection in SOCS5 overexpressing microglial cells and found reduced levels of pro-inflammatory cytokines (IL-6 and TNF-α) post JEV infection (Supplementary Fig. 3B,D). This resulted into enhanced viral replication in SOCS5 overexpressing cells (Supplementary Fig. 1B,C). In addition, we also checked the levels of miR-432 in JEV infected mice brain and we found decreased levels of miR-432 in brain of 2 day and 4 day infected mice. The seed sequence present in mature miR-432 of mouse and human are conserved, therefore we checked the levels of SOCS5 in JEV infected mice brain by western blotting and immunohistochemistry. We observed elevated levels of SOCS5 upon JEV infection in mice brain tissue. These findings support that JEV downregulates miR-432 in order to induce SOCS5 expression, to negatively regulate the Jak-STAT pathway to aid the replication and survival of the virus.

Cells trigger various anti-viral mechanisms to combat viral infection. The effect of JEV on the expression pattern of miR-432 is time dependent. Further study is required to understand the expression of miR-432 in the *in vivo* conditions at later time points. The successful establishment of JEV in the CNS takes place due to various strategies employed by the virus for suppressing the cellular immune response further resulting into neuroinflammation. The study of microRNAs in modulating expression of immune regulatory genes will render a better understanding of viral pathogenesis.

## Materials and Methods

### Cell Culture

Human microglial cells (CHME3) were cultured in Complete Dulbecco Modified Eagle Medium (DMEM) (#12100-046, Gibco, Rockville, MD,USA) with 10% heat inactivated Fetal Bovine Serum (16000-044; Gibco BRL) and 100 U Penicillin and 100 μg/ml Streptomycin (#10378016; Gibco-BRL). Porcine kidney cells (PS cells) were used for JEV Plaque assay to determine JEV titre and C6/36 cells were used for JEV propagation as described elsewhere^10^.

### Animal experiments and JEV infection

JEV (JaOArS982 strain) was a kind gift from Dr. Anirban Basu, National Brain Research Centre (NBRC) Manesar, India. All animal experiments were conducted at NBRC, Manesar, India. The experiments were performed according to the protocol approved by the institutional animal ethics committee of the National Brain Research Centre (NBRC) Manesar, India. 3 to 4 days old BALB/c mice of either sex were injected with 100 pfu of JE virus (strain JaOArS982) intracerebrally. Control animals were injected with equal amount of PBS. JEV infected mice were sacrificed at 2 and 4 days post-infection and control mice were sacrificed at 4 days post-infection. The MOI of 5 has been used to infect CHME3 cells by JEV (strain JaOArS982). Mock infected cells were used as control. Cells were harvested at 24 and 48 hours post-JEV infection for RNA isolation and western blotting. The methods were carried out in “accordance” with the approved guidelines.

### Overexpression and silencing of Anti-miR-432

CHME3 cells were transfected in 6 well plates with 100 pmol of miR-432 seed sequence mimic (Bioserve, Hyderabad, India) by using Lipofectamine 2000 (#11668-019; Invitrogen) according to manufacturer’s protocol. Scrambled seed sequence of miR-432 was transfected as control. miR-432 oligo and scramble sequence have been mentioned in Table 1. To silence miR-432, 100 pmol of anti-miR-432 (# AM17000, Ambion) was transfected and JEV infection was given 24 hours post transfection. 100 pmol Cy3 labelled scrambled Anti-miR (#AM17011; Ambion) was used as negative control. The overexpression and silencing of miR-432 was confirmed by real time PCR using miR-432 specific TaqMan primer and probe (# 4427975, Ambion). The cells were harvested after 48 hours post transfection.

### SOCS5 knock down

10 nM of siRNA specific to SOCS5 (#SR306430, Origene) was transfected into CHME3 cells and JEV infection (MOI 5) was given 30 hours post transfection. Cells were harvested 24 hours post JEV infection. The knock down of SOCS5 was confirmed by western blotting.

### RNA isolation and real Time PCR

miRNeasy kit (#217004; Qiagen) was used for miRNA isolation from harvested cells. Multiscribe TaqMan reverse transcriptase (#4366596; Applied Biosystems) was used to make cDNA with miR-432 specific primers. Real time PCR (ABI VII A7 RT-PCR) was done by using miR-432 specific TaqMan probe (# 4427975, Ambion) and universal PCR master mix (#4324018; Applied Biosystems). The expression of miR-432 was normalized by RNU24 expression as endogenous control.

To estimate the viral RNA, total RNA was extracted from cells by using RNeasy mini kit (#74106, Qiagen) according to manufacturer’s protocol. cDNA was synthesized by using superscript II (#11904-018, Invitrogen) according to manufacturer’s protocol. Viral RNA was isolated from culture supernatant by using High pure viral RNA kit (#11858882001, Roche). The primers used in this study have been mentioned in the Table 1. To extract the RNA from mice brain tissue, the tissue was homogenized and RNA was extracted using miRNeasy kit (# 217004; Qiagen). cDNA was synthesized and miR-432 levels were determined by real time PCR. RNU-6 was used as normalization control.

### Cloning of human SOCS5 gene

To overexpress SOCS5 gene in CHME3 cells, SOCS5 gene was cloned into eukaryotic vector pcDNA 3.1 vector. The primers used to amplify SOCS5 from human genomic DNA are listed in Table 1. Overexpression of SOCS5 was confirmed by western blotting.

### Western Blotting

Cell pellet was lysed in RIPA buffer (50 mM Tris-HCl, pH 7.5, 150 mM NaCl 1% NP-40, 0.1% SDS, 0.5% sodium deoxycholate) with 1X proteCEASE-50 (#427P;G-Biosciences, St.Louis, MO,USA) 1μM PMSF. Protein was quantified by Bicinchoninic acid (BCA) assay. The protein was resolved on SDS-PAGE and transferred on PVDF membrane. Membrane was kept for blocking in 5% skimmed milk in 1X TBS-Tween20. Thereafter, the membrane was incubated in primary antibody (1:1000) overnight. HRP-conjugated secondary antibody incubation was used for 1 hour and then membrane was washed thrice with 1X TBST and developed by using Super-Signal developing reagent as HRP substrate. The anti-STAT1 (#9172 Cell Signaling technology), anti-phospho-Y701-STAT1 (#9167 Cell Signaling technology), anti-SOCS-5 (#sc-5607, Santa cruz biotechnology) anti-JEV NS1 (#ab41651; Abcam) and anti-β-tubulin (#ab6046; Abcam) primary antibodies were used after diluting in 5% BSA in TBST buffer. HRP-conjugated anti-rabbit (#ab6721-1; Abcam) and anti-mouse (#ab97046, Abcam) secondary antibody was used in 1:50,000 dilutions.

### MicroRNA target prediction and validation

Various bioinformatics tools like Pictar (MDC, Berlin, Germany), Target Scan 5.2 (Whitehead Institute of Biomedical Research, MIT, Boston, USA) and MicroRNA.org were used to predict SOCS5 3′UTR as potential target of miR-432. The binding site in 3′UTR of SOCS5 was identified by using Human Target Scan.

For validation of microRNA targeting, 3′UTR clone of SOCS5 in pEZX-MT06 luciferase vector (#HmiT054032-MT06, GeneCopoeia) was transfected into HeLa cells along with miR-432 mimic or scramble mimic to observe the effect on luciferase activity. To generate the mutant clone, the binding site of miR-432 present in SOCS5 3′UTR (2242–2249) was deleted by using site directed mutagenesis. The mutated fragments were joined by using recombination PCR. Primers used for mutant generation and recombination PCR have been listed in Table 1. The mutated 3′UTR was cloned in pEZX-MT06 vector and transfected into HeLa cells along with miR-432 mimic to check luciferase activity and β-Galactosidase (700 ng) vector was co-transfected as normalization control.

### Luciferase assays

CHME3 cells were transfected with ISRE Luciferase reporter plasmid (1 μg) or along with miR-432 mimic, scrambled or anti-miR-432 (100 pmol). β-Galactosidase (700 ng) vector was co-transfected as normalization control. Luciferase activity was measured 48 hours post-transfection. For infection, cells were counted 24 hours post transfection and infected with JEV (MOI 5). Cells were incubated for 24 hours after infection and lysed to measure luciferase activity. Luminescence activity was measured by lysing the cells in 1X lysis buffer provided by Luciferase Assay kit (#E4030; Promega, Madison, WI, USA) and luciferase assay reagent was added later as per manufacturer’s protocol. Perkin Elmer multiplate reader (Enspire 2300 Multimode plate reader) was used to measure luciferase activity. For normalization, β-Galactosidase activity was measured by using β-Galactosidase kit (#E2000; Promega, Madison, WI, USA) as per manufacturer’s protocol.

### Immunohistochemistry

Mock and JEV infected mice were sacrificed (2 days after infection) and their brains were excised. The brain tissue was fixed with 4% paraformaldehyde and 20 micro meter thick sections were prepared by using cryostat (Leica CM3050S) and mounted on slides. Antigen unmasking of sections was performed by boiling the sections dipped in Vector antigen unmasking solution (#H-3300, Vector Laboratories). The sections were incubated with anti-SOCS5 (#sc-5607, Santa cruz biotechnology) primary antibody at 4^o^C overnight. The sections were washed and incubated with Alexa 488 conjugated secondary antibody (A 11034, Life technologies) for 1 hour and washed. The sections were then mounted with mounting medium with DAPI (H-1200, Vector Laboratories) and viewed under florescent microscope (Zeiss Axio imager).

### ELISA of TNF-α

CHME3 cells were transfected by 100 pmol of miR-432 mimic. 24 hours post transfection, JEV infection was given. Scramble sequence was used as control. 24 hours post infection, the culture supernatant was collected and ELISA was performed by using human TNF-α ELISA kit (Cat No.-KHC3011, Invitrogen) according to manufacturer’s protocol.

### Statistical analysis

All experiments were conducted in triplicates and one-tailed, paired Student’s t-test was used to make comparison between data sets. Data was considered significant when P < 0.05; *denotes P < 0.05, **denotes P < 0.01, ***denotes P < 0.001

## Additional Information

**How to cite this article**: Sharma, N. *et al*. Japanese Encephalitis Virus exploits the microRNA-432 to regulate the expression of Suppressor of Cytokine Signaling (SOCS) 5. *Sci. Rep.* **6**, 27685; doi: 10.1038/srep27685 (2016).

## Supplementary Material

Supplementary Information

## Figures and Tables

**Table 1:** List of Primers and MicroRNA Oligos.

Viral NS3 Forward, 5′ AGAGCGGGGAAAAAGGTCAT 3′
Viral NS3 Reverse, 5′ TTTCACGCTCTTTCTACAGT 3′
GAPDH Forward, 5′ ATGGGGGAAGGTGAAGGTCG 3′
GAPDH Reverse, 5′ GGGGTCATTGATGGCAACAATA 3′
SOCS5-UTR Forward, 5′ AACTCGAGACT CTC CGG TCC CCA AAG 3′
SOCS5-UTR Reverse, 5′ AA GCGGCCGCTCA ACT TCT AAA GAA ATT C 3′
IFIT-1 Forward, 5′ AGAAGCAGGCAATCACAGAAAA 3′
IFIT-1 Reverse, 5′ CTGAAACCGACCATAGTGGAAAT 3′
IFIT-2 Forward, 5′ CACATGGGCCGACTCTCAG 3′
IFIT-2 Reverse, 5′ CCACACTTTAACCGTGTCCAC 3′
IL-6 Forward, 5′ ACTCACCTCTTCAGAACGAATTG 3′
IL-6 Reverse, 5′CCATCTTTGGAAGGTTCAGGTTG 3′
SOCS5-UTR MUTANT Forward, 5′ GAA ACT TGA AAA AGC ACT TGA TG 3′
SOCS5-UTR MUTANT Reverse, 5′ CAT CAA GTG CTT TTT CAA GTT TC 3′
SOCS5 Forward, 5′ CCCTCGAGATGGATAA AGTGGGAAAA ATG 3′
SOCS5 Reverse, 5′ ACCGGATCCTTACTTTGCCTTGACTGG 3′
miR-432 mimic sequence, 5′ UCUUGGAGUAGGUCAUUGGGUGG 3′
miR-432 scramble sequence, 5′ CAT CAA GTG CTT TTT CAA GTT TC 3′
