# Supplementary material for: Japanese Encephalitis Virus exploits the microRNA-432 to regulate the expression of Suppressor of Cytokine Signaling (SOCS) 5
Source: Sci Rep. 2016 Jun 10;6:27685. doi: 10.1038/srep27685 (PMC4901348; doi:10.1038/srep27685)

Japanese Encephalitis Virus exploits the microRNA-432 to regulate the expression of Suppressor of Cytokine Signaling (SOCS) 5

Nikhil Sharma[1], Kanhaiya L. Kumawat[2], Meghana Rastogi[3], Anirban Basu[2], Sunit K. Singh[3]

Supplementary Figure1: SOCS5 overexpression upregulates JEV replication

SOCS5 was cloned in eukaryotic expression vector pcDNA 3.1 and transfected into CHME3 cells to determine the effect of SOCS5 overexpression on JEV replication. **(A)** Overexpression of SOCS5 was confirmed by western blotting. The average fold change values w.r.t. to control have been mentioned. **(B)** Graph showing increased viral RNA in SOCS5 overexpressed cells. Real time PCR was done by using viral NS3 specific primers. GAPDH was used for normalization. Fold change was calculated by $2^{-\Delta\Delta C}{}_t$ method. **(C)** Western blot image of viral NS1 protein showing enhanced viral protein upon SOCS5 overexpression. The average fold change values with respect to control have been mentioned. The data are shown as mean ± S.E from three independent experiments.

Supplementary Figure 2: JEV suppresses host immune response

The replication of JEV was checked by Real time PCR and effect of JEV infection on STAT1 phosphorylation was analysed. **(A)** CHME3 cells were infected by JEV (MOI 5) and JEV replication was checked at 24 and 48 hours post infection by using JEV NS3 specific primers. GAPDH was used for normalization. **(B)** Real time PCR graph depicting JEV replication in mice brain tissue. BALB/c mice pups were infected by JEV (100 pfu) and harvested after 2 day and 4 day post infection. **(C)** Real time PCR graph showing IFIT-1 levels in 2 day and 4 day JEV infected mice brain tissue. For statistical analysis 4 day JEV sample was compared to 2 day JEV sample. **(D)** Real time PCR graph showing IFIT-2 levels in 2 day and 4 day JEV infected mice brain tissue. For statistical analysis 4 day JEV sample was compared to 2 day JEV sample. **(E)** Western blots showing enhanced STAT1 phosphorylation in 2 day JEV infected mice brain. STAT1 phosphorylation reduces in 4 day infected sample as compared to 2 day infected sample. The average fold change values with respect to control have been mentioned. **(F)** Densitometry graph showing STAT1 phosphorylation in 2 day and 4 day infected mice. Phospho / total STAT1 ratio was calculated which was divided by β-tubulin values for normalization. Fold change was calculated with respect to uninfected control. The data are shown as mean ± S.E from three independent experiments.

Supplementary Figure 3: miR-432 overexpression enhances cellular immune response

miR-432 was overexpressed in CHME3 cells and JEV infection was given after 24 hours. Levels of pro-inflammatory cytokines were analysed. **(A)** Real time PCR graph showing increased IL-6 levels in miR-432 overexpressing JEV infected cells. **(B)** Real time PCR graph showing decreased IL-6 levels in SOCS5 overexpressing JEV infected cells. **(C)** ELISA of TNF-α from culture supernatants of miR-432 overexpressing JEV infected cells. **(D)** ELISA of TNF-α from culture supernatants of SOCS5 overexpressing JEV infected cells. The data are shown as mean ± S.E from three independent experiments.

Supplementary Figure-1: SOCS5 overexpression upregulates JEV replication

A

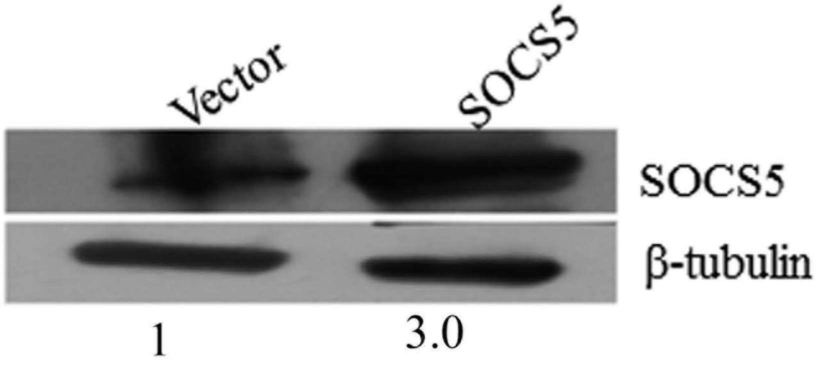

B

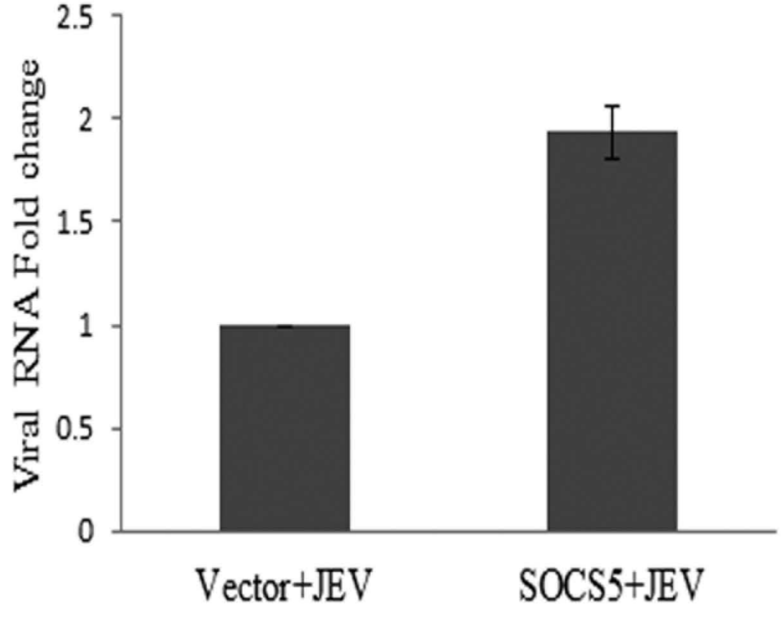

C

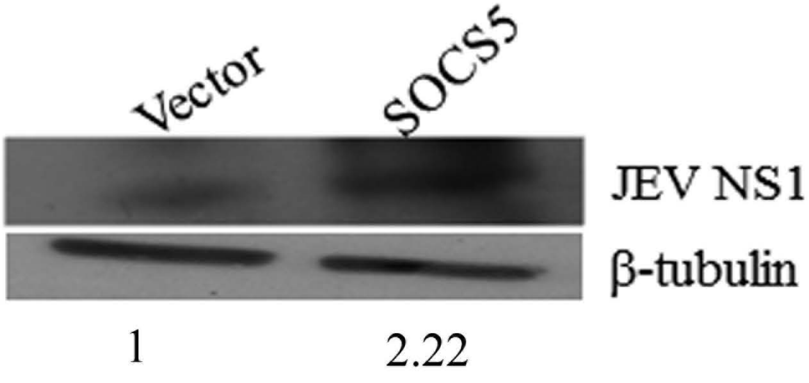

Supplementary Figure-2: JEV suppresses host immune response

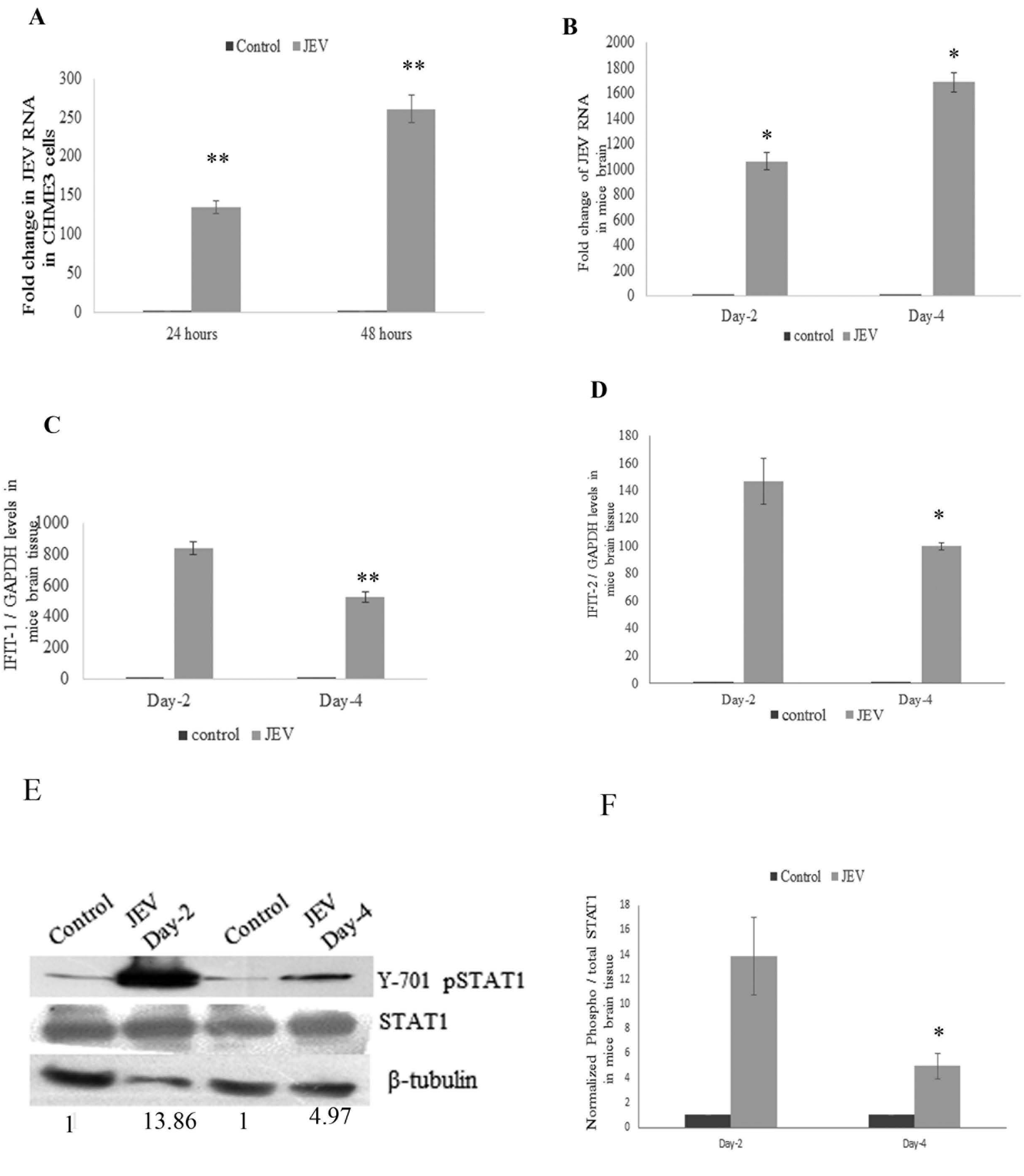

Supplementary Figure-3: miR-432 overexpression enhances cellular inflammatory response

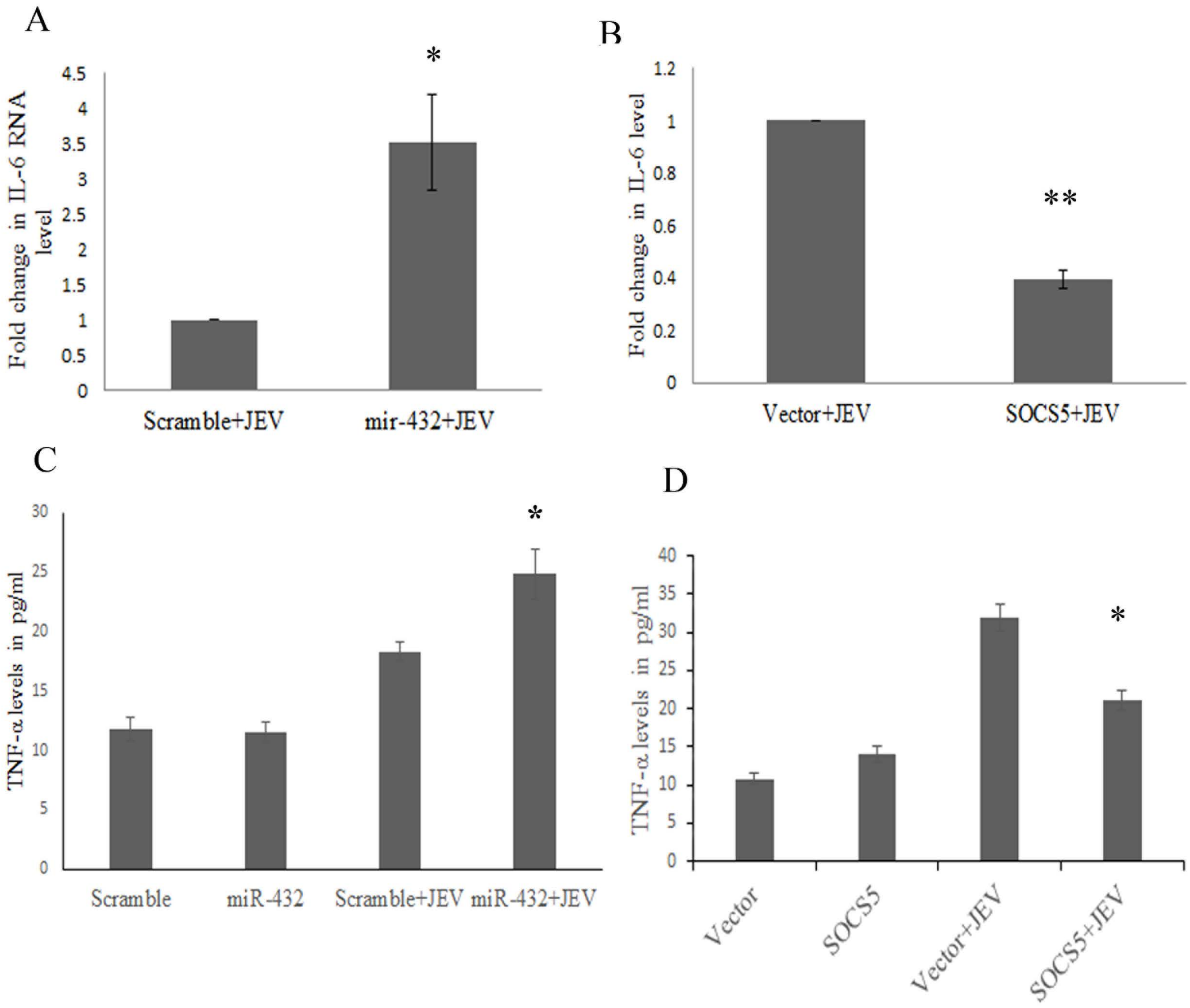

Supplement: Supplementary Information [file srep27685-s1.pdf]
